# Effect of biological, psychological, and social factors on maternal depressive symptoms in late pregnancy: a cross-sectional study

**DOI:** 10.3389/fpsyt.2023.1181132

**Published:** 2023-06-06

**Authors:** Xu Chen, Meilin Liu, Fanli Min, Jiao Tong, Yuan Liu, Qian Meng, Teng Zhang

**Affiliations:** Department of Obstetrics, Lianyungang Maternal and Child Health Hospital, Lianyungang, Jiangsu, China

**Keywords:** depressive symptoms, pregnancy, biological factors, psychological factors, social factors

## Abstract

**Introduction:**

Depression commonly occurs during pregnancy and has become a major public health concern. Depression not only affects the individual but also causes adverse consequences for families and children. However, little is known regarding the depression status and its influencing factors in women during late pregnancy in China. This study aimed to assess the prevalence of maternal depressive symptoms in late pregnancy during the coronavirus disease 2019 (COVID-19) pandemic and further explore the effect of biological, psychological, and social factors on depressive symptoms.

**Methods:**

An institution-based cross-sectional survey was conducted among eligible women in the late pregnancy stage and underwent prenatal examination at Lianyungang Maternal and Child Health Hospital in Jiangsu Province, Eastern China from December 2022 to February 2023. Data regarding depressive symptoms and biological, psychological, and social factors of the pregnant women were collected via a structured questionnaire. Chi-square test, Fisher's exact tests, and binary logistics regression were used to analyze the data.

**Results:**

In total, 535 women in the late pregnancy stage were included in this study, 75 (14.0%) of whom exhibited depressive symptoms. A binary logistic regression analysis revealed that pregnant women who were multiparous (*OR*: 2.420, 95% *CI*: 1.188–4.932) and had moderate or severe insomnia symptoms (*OR*: 4.641, 95% *CI*: 1.787–12.057), anxiety (*OR*: 8.879, 95% *CI*: 4.387–17.971), high fear of COVID-19 (*OR*: 2.555, 95% *CI*: 1.255–5.199), moderate or severe family dysfunction (*OR*: 2.256, 95% *CI*: 1.141–4.461), and poor social support (*OR*: 2.580, 95% *CI*: 1.050–6.337) tended to show depressive symptoms. Conversely, pregnant women who received regular prenatal care (*OR*: 0.481, 95% *CI*: 0.243–0.951) and had good drinking water quality at home (*OR*: 0.493, 95% *CI*: 0.247–0.984) were more likely to avoid developing depressive symptoms.

**Conclusion:**

This study found that the prevalence of maternal depressive symptoms during late pregnancy was high and had multiple influencing factors. Thus, screening for depressive symptoms in women in the late pregnancy stage and providing special intervention programs are necessary, especially for those with risk factors.

## Introduction

Depression is a common mental health problem worldwide, affecting >30 million people of all ages (1). Pregnant women are at an especially high risk of developing depression (2). During pregnancy, almost all women experience different degrees of psychological disorders, including depression, due to specific physiological cycles and changes in hormone levels (3, 4). Previous studies have reported that the incidence of prenatal depression in pregnant women varies from 4.4 to 76.1% and that depression is a major public health concern worldwide (5, 6). Notably, the coronavirus disease 2019 (COVID-19) pandemic has seriously affected people's lives and added numerous stressors to pregnant women, thereby increasing the prevalence of depression in them (7, 8). A recent systematic review and meta-analysis reported that the prevalence of depression among pregnant women during the pandemic was 25.6% (9). A study performed in the United States indicated that more than a third of pregnant women reported having depression during the pandemic (10). An Italian study reported that 45.7% of pregnant women experienced clinically relevant perinatal depression during the pandemic (11). Studies performed in China have demonstrated that 6.9–36.1% of pregnant women exhibited depressive symptoms during the COVID-19 pandemic (12, 13). However, the current management patterns are inadequate for diagnosing and treating most pregnant women with depressive symptoms.

Without timely intervention, prenatal depression and other psychological disorders tend to persist, thereby adversely affecting maternal outcomes and child development (14). Reportedly, prenatal depression is associated with an increased risk of adverse pregnancy outcomes, such as miscarriage, preterm birth, and lower birth weight (15). Maternal depression may affect child outcomes by altering placental function, causing epigenetic changes in children, and triggering stress responses (16, 17). In addition, pregnant women with comorbid depression exhibited lower levels of exercise management, diet management, and management goals, which can lead to weight-management problems in pregnant women, affecting various maternal and child health outcomes (18). In some cases, depressed pregnant women even commit suicide, which can seriously affect families and society (19). Therefore, it is crucial to identify the potentially controllable risk factors of depression among pregnant women and implement early and effective prevention programs.

The bio-psycho-social medicine model emphasizes that in addition to biological factors, psychological and social factors affecting human health should also be considered (20, 21). Previous research regarding maternal depression during the COVID-19 pandemic has revealed that several biological, psychological, and social factors are associated with maternal depression. The biological factors include cervical insufficiency and a history of psychiatric illness (8, 19) and the psychological factors include psychological preparation for pregnancy, anxiety, pregnancy stress, and fear related to COVID-19 (1, 11). The social factors include occupation, total monthly income, living status, pregnancy knowledge, intimate partner violence, and critical health literacy (1, 12, 22). However, most studies have simply analyzed the influencing factors of depression in pregnant women from several aspects, and a more comprehensive analysis is needed.

The identification of risk factors is important for the development of effective interventions. To provide better-targeted and correct intervention programs for pregnant women, it is necessary to gain a clear understanding of the status of depressive symptoms and the factors that affect them. With the progression through each pregnancy stage, pregnant women may exhibit varying levels of depression in different pregnancy cycles, and different interventions should be adopted in different periods to reduce the occurrence of depressive symptoms. Studies performed in Turkey have shown that more pregnant women develop depressive symptoms in their third trimester (23). However, the magnitude and predictors of maternal depressive symptoms in late pregnancy have not been adequately studied. In addition, mental health indicators, such as depression, in pregnant women differ significantly between countries (14). The influencing factors of maternal depressive symptoms in late pregnancy may also vary from country to country or even from region to region. Different regions need to establish health care policies that meet the unique psychological needs of women in late pregnancy in their respective regions (14). Although similar studies have been performed in several countries and regions, no such research has been reported in the northeast area of Jiangsu Province, Eastern China. Therefore, this study investigated the maternal depressive symptoms in late pregnancy in Lianyungang, Jiangsu Province, Eastern China during the COVID-19 pandemic. Concomitantly, based on the bio-psycho-social model, the influencing factors of maternal depressive symptoms in late pregnancy were analyzed from three aspects: biological factors, psychological factors, and social factors. This study will provide a scientific basis for reducing maternal depression symptoms in late pregnancy.

## Materials and methods

### Study design and setting

An institution-based cross-sectional survey was conducted from December 2022 to February 2023 at the Lianyungang Maternal and Child Health Hospital in Jiangsu Province, Eastern China. Lianyungang, also known as Port City, is located in the northeast region of Jiangsu Province, with three districts and three counties under its jurisdiction, covering a total area of 7,615 km^2^. At the end of 2021, the permanent population of Lianyungang was 4.602 million people, and in 2022, the gross production value was 400.5 billion yuan. Lianyungang Maternal and Child Health Hospital is the only national third-grade class-A maternal and child health hospital in Lianyungang; it covers a total floor area of ~92,720.3 m^2^. It currently has 938 beds and is responsible for providing life-cycle health care services for women and children in the city.

### Participants

The subjects of this study were women in late pregnancy who underwent prenatal examination at the Department of Obstetrics and Gynecology of Lianyungang Maternal and Child Health Hospital in Jiangsu Province, Eastern China, between December 2022 and February 2023. A structured questionnaire was distributed to women in late pregnancy who met the inclusion criteria and were waiting for an examination in the outpatient clinic. The inclusion criteria were as follows: (1) a definite diagnosis of pregnancy with a gestational period of ≥28 weeks (gestational weeks calculated from the expected delivery date and the survey date); (2) age ≥18 years; (3) the ability to understand the research content and follow the research process; and (4) consent to participate in this study. The exclusion criteria were as follows: (1) intellectual disability or cognitive impairment; (2) serious diseases not related to pregnancy; (3) inability to communicate normally; (4) lack of consent to participate in this study. The questionnaire was completed independently by pregnant women. Before filling out the questionnaire, the investigators explained the purpose, content, and related benefits and risks of the study to the pregnant women. The questionnaire was collected by five investigators with uniform training. In total, 550 questionnaires were distributed in this study, of which 15 were excluded because of incomplete filling. Finally, 535 valid questionnaires were collected in this study, yielding an effective rate of 97.3%.

### Data collection

A structured questionnaire designed by reading the literature and consulting experts was used for data collection. Before the formal survey, we conducted a pre-survey in the research institutions and improved the questionnaire to ensure its validity. The questionnaire consisted of four parts: depression symptoms, biological factors, psychological factors, and social factors. The biological factors included age, parity, focus on nutrition intake during pregnancy, threatened abortion, gestational diabetes mellitus, and experience with colds since the beginning of the pregnancy. The psychological factors included the planning of the pregnancy, perceived poor physical resistance (referring to the perception that one is more susceptible to illness than others), perceived stress, insomnia symptoms, anxiety symptoms, and experience of COVID-19 fear. Finally, the social factors included employment status during pregnancy, residence, education level, monthly family income, delays in prenatal care, residential noise, quality of the drinking water at home, knowledge of pregnancy, family function, and social support.

Depressive symptoms were assessed using the Patient Health Questionnaire-9 (PHQ-9) (24), which is a simple and effective self-report questionnaire that is used as an auxiliary diagnostic tool for depression. The PHQ-9 has good reliability and validity in evaluating the severity of depressive symptoms of respondents (25). The questionnaire consists of nine items, each of which is rated on a 4-point Likert scale, ranging from 0 (not at all) to 3 (almost every day). The total score ranges from 0 to 27, with higher scores indicating more severe depressive symptoms. A score of 0–4 is considered as indicating the absence of depression, 5–9 indicates mild depression, 10–14 indicates moderate depression, and 15–27 denotes severe depression (25). The critical significant depression score recommended by this questionnaire is 10 (26). Therefore, a cut-off value of 10 points was adopted in the present study. The PHQ-9 has been widely used to assess depressive symptoms in pregnant women in many countries, including China, and has high internal consistency (1, 12, 27, 28). Herein, the Cronbach's α was 0.857.

Insomnia symptoms were assessed using the Insomnia Severity Index (ISI) scale, which is employed to screen for insomnia symptoms and assess their severity (27). This scale is a handy tool comprising seven items, each of which is scored using a 5-point Likert scale ranging from 0 to 4. The total score is the sum of seven items and ranges from 0 to 28, with higher scores indicating more severe insomnia symptoms. The total scores range from 0 to 7 for no insomnia, 8 to 14 for mild insomnia, and 15 to 28 for moderate or severe insomnia (29). This scale has been widely utilized in epidemiological surveys and exhibits good reliability and validity (30). Herein, the Cronbach's α was 0.897.

Anxiety symptoms were assessed using the Generalized Anxiety Disorder-7 (GAD-7) scale (31), which comprises seven items and was used to measure the severity of anxiety symptoms in the respondents. A 4-point Likert scale was used to rate each item, ranging from 0 (not at all) to 3 (almost every day). The total scores range from 0 to 21, with higher scores indicating higher levels of anxiety symptoms. Those who score ≥7 were considered to have anxiety symptoms (12). GAD-7 has been validated in multiple populations, including pregnant women (12, 27, 31). Herein, the Cronbach's α was 0.934.

Experience of COVID-19 fear was measured using the Fear of COVID-19 Scale (FCV-19S) (32). The scale is a 7-item self-assessment questionnaire used to measure emotional fear occurring during the COVID-19 pandemic. Each item is rated on a 5-point Likert scale ranging from 1 (strongly disagree) to 5 (strongly agree), with higher scores indicating higher levels of fear. Pregnant women with scores >21 were considered to have a high fear of COVID-19 (33). The scale has been validated in many countries with good reliability and validity (33, 34). Herein, the Cronbach's α was 0.892.

Family function was measured using the Family APGAR questionnaire developed by Smilkstein (35). The aim of this questionnaire is to assess satisfaction with social support received from family members based on adaptability, partnership, growth, affection, and resolve. Each item of the questionnaire is rated on a 3-point Likert scale ranging from 0 (hardly ever) to 2 (almost always). The total score is calculated by summing the scores for each item, with a minimum score of 0 and a maximum score of 10; higher scores indicate better family functioning. Family functioning was divided into two levels, 0–6 indicated a moderately or severely dysfunctional family and 7–10 indicated a highly functional family (35). The Family APGAR questionnaire is widely used to evaluate family function among pregnant women and exhibits good reliability and validity (12). Herein, the Cronbach's α was 0.917.

Social support was assessed using the Oslo 3-item social support scale, which comprises three items and is often used to assess social support–related issues in community settings (36). This scale evaluates the number of close people on whom individuals can rely when facing serious problems and their level of concern and the level of ease of obtaining practical help from neighbors. Each item score is summed to obtain an overall score, with a minimum of 3 and maximum of 14; higher scores indicate higher levels of social support. The total score ranges from 3 to 8 for poor support and from 9 to 14 for moderate or strong support (36). Herein, the Cronbach's α was 0.60.

### Ethical approval

The Ethics Committee of Lianyungang Maternal and Child Health Hospital approved the study. Written informed consent was obtained from all study subjects. All subjects were also assured that their data would be kept confidential. The whole study procedure conformed to the criteria of the 1964 Declaration of Helsinki and its later amendments.

### Data processing and analysis

The valid questionnaires with no missing items were coded and entered into a database established by Epidata3.1 (EpiData Association, Odense, Denmark) software via double entry, to ensure the accuracy of the data. A total of 535 pregnant women with complete data were included in the study. Biological, psychological, and social factors for inclusion in the study were selected based on the Directed Acyclic Graphs (DAG) (37). The SPSS 21.0 (IBM Corporation, Armonk, State of New York) software was used to analyze the data. Continuous data were described as the mean and standard deviation (SD), whereas categorical data were described as frequency and percentage. A bivariate analysis was performed, and chi-squared test and Fisher's exact tests were used to evaluate the differences in depressive symptoms among the different groups. The statistically significant variables in the bivariate analysis were included in the binary logistic regression model, to evaluate the independent effect of each variable after excluding confounding factors. The presence or absence of depressive symptoms was the dependent variable. In the regression analysis, 75 pregnant women had depressive symptoms. Before conducting the binary logistic regression, we performed a collinearity test and found that the variance inflation factor of each predictor variable was <10 and that the tolerance was much >0.1. This indicates an absence of collinearity between predictor variables. All tests were performed with a two-tailed statistical test, and significance was set at a *P*-value of <0.05.

## Results

### Current status of maternal depressive symptoms during late pregnancy

**Figure 1:**
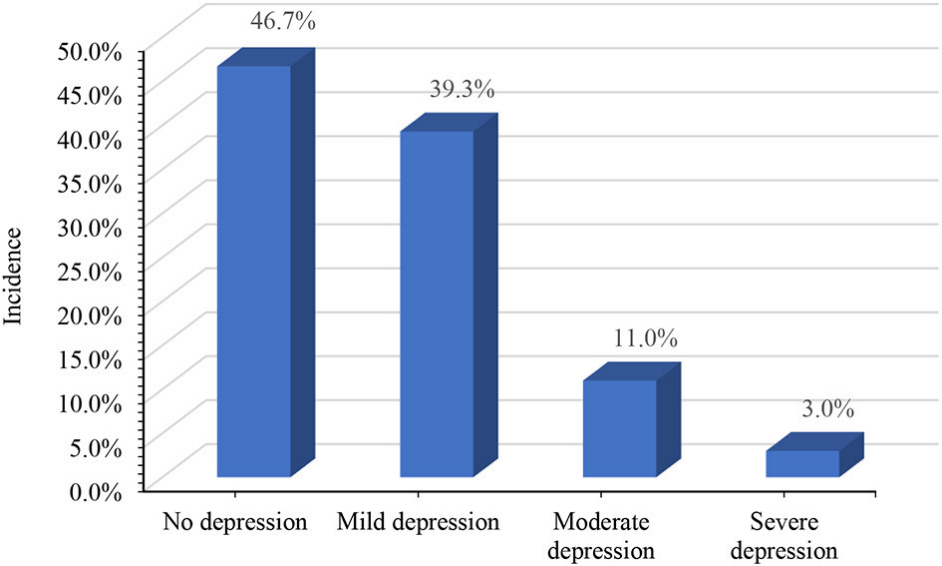
Current status of maternal depressive symptoms during late pregnancy.

Among the 535 women in the late pregnancy stage included in this study, 210 (39.3%) exhibited mild depressive symptoms, 59 (11.0%) exhibited moderate depressive symptoms, and 16 (3.0%) exhibited severe depressive symptoms according to the scores of the PHQ-9 scale. Using the recommended cutoff value of 10 points, the incidence of depressive symptoms was 14.0% in our study (Figure 1).

### Biological factors

The mean age of 535 women in the late pregnancy stage was 29.39 years (SD = 4.47 years). Approximately half these pregnant women (44.3%) were giving birth for the first time. A large number of pregnant women (67.3%) paid great attention to nutrition intake during pregnancy, and almost one fifth of them (18.9%) had experienced threatened abortion. A small number of pregnant women (11.6%) had gestational diabetes mellitus and more than half (62.2%) had experienced colds since the beginning of the pregnancy. The bivariate analysis revealed that parity, focus on nutrition during pregnancy, and threatened abortion were significantly associated with depressive symptoms (*P* < 0.05; Table 1).

**Table 1:** Depressive symptoms by biological factors.

**Variable**	**Total *n* (%)**	**Depressive symptoms** ***n*** **(%)**		***P***
		**No**	**Yes**	
**Age group (years)**				
<35	468 (87.5)	405 (86.5)	63 (13.5)	0.327
≥35	67 (12.5)	55 (82.1)	12 (17.9)	
**Parity**				
Primiparity	237 (44.3)	215 (90.7)	22 (9.3)	**0.005**
Multiparity	298 (55.7)	245 (82.2)	53 (17.8)	
**Focus on nutrition during pregnancy**				
Yes	360 (67.3)	322 (89.4)	38 (10.6)	**0.001**
No	175 (32.7)	138 (78.9)	37 (21.1)	
**Threatened abortion**				
Yes	101 (18.9)	78 (77.2)	23 (22.8)	**0.005**
No	434 (81.1)	382 (88.0)	52 (12.0)	
**Gestational diabetes mellitus**				
Yes	62 (11.6)	50 (80.6)	12 (19.4)	0.198
No	473 (88.4)	410 (86.7)	63 (13.3)	
**Colds during pregnancy**				
Yes	333 (62.2)	284 (85.3)	49 (14.7)	0.552
No	202 (37.8)	176 (87.1)	26 (12.9)	

Significant values are in bold.

### Psychological factors

Among the 535 pregnant women studied here, nearly a quarter (23.9%) felt that their current stress level was high. In turn, < $\frac{1}{2}$ of them (40.2%) had no insomnia symptoms, whereas more than one in five (20.2%) had symptoms of anxiety and nearly one in three (27.7%) had a high fear of COVID-19. The bivariate analysis found that perceived poor resistance, perceived stress, insomnia symptoms, anxiety symptoms, and experience of COVID-19 fear were significantly correlated with depressive symptoms (*P* < 0.05; Table 2).

**Table 2:** Depressive symptoms by psychological factors.

**Variable**	**Total *n* (%)**	**Depressive symptoms** ***n*** **(%)**		***P***
		**No**	**Yes**	
**Planning of pregnancy**				
Planned	331 (61.9)	285 (86.1)	46 (13.9)	0.918
Unplanned	204 (38.1)	175 (85.8)	29 (14.2)	
**Perceived poor resistance**				
Agree	26 (4.9)	16 (61.5)	10 (38.5)	**0.001** ^*^
Disagree	509 (95.1)	444 (87.2)	65 (12.8)	
**Perceived stress**				
Low	407 (76.1)	369 (90.7)	38 (9.3)	**<0.001**
High	128 (23.9)	91 (71.1)	37 (28.9)	
**Insomnia symptoms**				
No insomnia	215 (40.2)	205 (95.3)	10 (4.7)	**<0.001**
Mild insomnia	246 (46.0)	214 (87.0)	32 (13.0)	
Moderate or severe insomnia	74 (13.8)	41 (55.4)	33 (44.6)	
**Anxiety symptoms**				
Yes	108 (20.2)	57 (52.8)	51 (47.2)	**<0.001**
No	427 (79.8)	403 (94.4)	24 (5.6)	
**Experience of COVID-19 fear**				
Low	387 (72.3)	350 (90.4)	37 (9.6)	**<0.001**
High	148 (27.7)	110 (74.3)	38 (25.7)	

COVID-19, Corona Virus Disease 2019.

^*^Means that the theoretical number is too small, and the Fisher's exact test was used. Significant values are in bold.

### Social factors

Among the 535 pregnant women included in this study, nearly half (46.2%) were unemployed during their pregnancy. Furthermore, more than a third of these pregnant women (34.4%) did not attend their scheduled prenatal care because of the pandemic. A small number of pregnant women (10.3%) reported noise in their residence. More than half of the pregnant women (53.1%) stated that the quality of their drinking water at home was poor. Moreover, less than one third of the pregnant women (32.5%) had good knowledge about pregnancy, and a large number of pregnant women (69.0%) had a high family function. Finally, a small number of pregnant women (9.0%) claimed that they had poor social support. The bivariate analysis showed that prenatal care delay, residential noise, drinking water quality, knowledge of pregnancy, family function, and social support were significantly associated with depressive symptoms (*P* < 0.05; Table 3).

**Table 3:** Depressive symptoms by social factors.

**Variable**	**Total *n* (%)**	**Depressive symptoms** ***n*** **(%)**		***P***
		**No**	**Yes**	
**Employment status during pregnancy**				
Employed	288 (53.8)	249 (86.5)	39 (13.5)	0.731
Unemployed	247 (46.2)	211 (85.4)	36 (14.6)	
**Residence**				
Urban	357 (66.7)	312 (87.4)	45 (12.6)	0.182
Rural	178 (33.3)	148 (83.1)	30 (16.9)	
**Educational level**				
High school or below	179 (33.5)	152 (84.9)	27 (15.1)	0.615
College or above	356 (66.5)	308 (86.5)	48 (13.5)	
**Monthly family income**				
≤5,000 yuan	100 (18.7)	84 (84.0)	16 (16.0)	0.780
5,001–10,000 yuan	235 (43.9)	202 (86.0)	33 (14.0)	
≥10,001 yuan	200 (37.4)	174 (87.0)	26 (13.0)	
**Delays in prenatal care**				
Yes	184 (34.4)	150 (81.5)	34 (18.5)	**0.031**
No	351 (65.6)	310 (88.3)	41 (11.7)	
**Residential noise**				
Yes	55 (10.3)	41 (74.5)	14 (25.5)	**0.010**
No	480 (89.7)	419 (87.3)	61 (12.7)	
**Drinking water quality**				
Poor	284 (53.1)	229 (80.6)	55 (19.4)	**<0.001**
Good	251 (46.9)	231 (92.0)	20 (8.0)	
**Knowledge of pregnancy**				
Poor	145 (27.1)	116 (80.0)	29 (20.0)	**0.047**
Moderate	216 (40.4)	189 (87.5)	27 (12.5)	
Good	174 (32.5)	155 (89.1)	19 (10.9)	
**Family function**				**<0.001**
High function	369 (69.0)	338 (91.6)	31 (8.4)	
Moderate or severe dysfunction	166 (31.0)	122 (73.5)	44 (26.5)	
**Social support**				
Poor	48 (9.0)	30 (62.5)	18 (37.5)	**<0.001**
Moderate or strong	487 (91.0)	430 (88.3)	57 (111.7)	

Significant values are in bold.

### Binary logistic regression analysis of factors independently associated with maternal depressive symptoms in late pregnancy

The complete data obtained for the 535 pregnant women were analyzed by multivariate analysis. Using the presence or absence of depressive symptoms in the pregnant women as the dependent variable (with depression: 75 cases; without depression: 460 cases), 14 significant variables in the bivariate analysis (biological factors: parity, focus on nutrition during pregnancy, and threatened abortion; psychological factors: perceived poor resistance, perceived stress, insomnia symptoms, anxiety symptoms, and experience of COVID-19 fear; and social factors: delays in prenatal care, residential noise, drinking water quality, knowledge of pregnancy, family function, and social support) were included as independent variables in the binary logistic model, for analysis. The binary logistic regression analysis showed that biological factors such as parity affected the development of depression. In addition, the psychological factors that affected depression included insomnia symptoms, anxiety symptoms, and experience of COVID-19 fear. Finally, social factors such as delays in prenatal care, drinking water quality, family function, and social support were also affected depression. Pregnant women who were multiparous (*OR*: 2.420, 95% *CI*: 1.188–4.932), had moderate or severe insomnia symptoms (*OR*: 4.641, 95% *CI*: 1.787–12.057), had anxiety symptoms (*OR*: 8.879, 95% *CI*: 4.387–17.971), experienced high fear of COVID-19 (*OR*: 2.555, 95% *CI*: 1.255–5.199), had moderate or severe family dysfunction (*OR*: 2.256, 95% *CI*: 1.141–4.461), and had poor social support (*OR*: 2.580, 95% *CI*: 1.050–6.337) were more likely to have depressive symptoms. In contrast, pregnant women who had regular prenatal care (*OR*: 0.481, 95% *CI*: 0.243–0.951) and had good drinking water quality at home (*OR*: 0.493, 95% *CI*: 0.247–0.984) were more likely to avoid depressive symptoms (Table 4).

**Table 4:** Binary logistic regression analysis and predictors of depressive symptoms.

**Variable**	***OR***	**95% *CI***	***P***
**Parity**			
Primiparity	1		
Multiparity	2.420	1.188–4.932	**0.015**
**Focus on nutrition during pregnancy**			
Yes	1		
No	1.395	0.716–2.718	0.327
**Threatened abortion**			
Yes	1		
No	0.651	0.306–1.384	0.265
**Perceived poor resistance**			
Agree	1		
Disagree	0.549	0.169–1.784	0.318
**Perceived stress**			
Low	1.223	0.591–2.533	0.587
High	1		
**Insomnia symptoms**			
No insomnia	1		
Mild insomnia	2.004	0.878–4.576	0.099
Moderate or severe insomnia	4.641	1.787–12.057	**0.002**
**Anxiety symptoms**			
Yes	8.879	4.387–17.971	**<0.001**
No	1		
**Experience of COVID-19 fear**			
Low	1		
High	2.555	1.255–5.199	**0.010**
**Delays in prenatal care**			
Yes	1		
No	0.481	0.243–0.951	**0.035**
**Residential noise**			
Yes	1.244	0.499–3.102	0.640
No	1		
**Drinking water quality**			
Poor	1		
Good	0.493	0.247–0.984	**0.045**
**Knowledge of pregnancy**			
Poor	0.696	0.282–1.715	0.431
Moderate	0.982	0.435–2.217	0.964
Good	1		
**Family function**			
High function	1		
Moderate or severe dysfunction	2.256	1.141–4.461	**0.019**
**Social support**			
Poor	2.580	1.050–6.337	**0.039**
Moderate or strong	1		

Significant values are in bold.

## Discussion

This study assessed the incidence of maternal depressive symptoms in late pregnancy during the COVID-19 pandemic and explored the biological, psychological, and social factors risk factors of depressive symptoms. Herein, the detection rate of maternal depressive symptoms during late pregnancy was 14.0%. In a Chinese study using the same depression scale, the detection rate was lower than that recorded in Beijing (24.3%) (27) and higher than that reported in Shenzhen (6.9%) (12). Compared with studies that used the same depression assessment scale in other countries, the incidence of depressive symptoms in this study was comparable to that in Urumqi (13.6%) (1). This may be attributed to differences in study design, cultural context, economic conditions, and regions. Moreover, the COVID-19 pandemic posed a major threat to the mental health of pregnant women (14). The pandemic situation during the study period and the specific measures taken to contain the virus and mitigate its economic and social impact may also have contributed to the differences observed in the incidence of depression (38). The incidence of maternal depressive symptoms during late pregnancy in the current study was also much higher than that observed in the general Chinese population (2.1%) (18). The results of the current study indicate that women in the late pregnancy stage exhibit a higher risk of depression and that the early identification of depression symptoms and appropriate mental health interventions are urgently warranted to protect the mental health of these women. In practice, screening for psychological symptoms should be added to prenatal care and psychological support and intervention programs should be provided for those with positive symptoms. This study found that the biological factor of parity was associated with depression alongside psychological factors, such as insomnia and anxiety symptoms, and experience of COVID-19 fear. Furthermore, social factors such as delays in prenatal care, drinking water quality, family function, and social support were associated with the development of depressive symptoms.

### Effect of biological factors on depression

Previous studies have shown that maternal depression is significantly related to parity, with higher scores of depression among multiparous women, which is consistent with our study (39). Our study indicated that multiparous women had a higher probability of developing depression than did primiparous women. Another study found that women with more than three daughters were almost four times more likely to suffer from depression (40). This may be attributed to multiple adverse obstetric experiences in the past and the stress of caring for existing children. Therefore, a greater consideration of multiparity is needed iPn psychological interventions for pregnant women and related policy formulation.

### Effect of psychological factors on depression

A meta-analysis reported that ~45.7% of pregnant women experienced sleep disorders during pregnancy (41). Our study found that 59.8% of women in the late pregnancy stage exhibited insomnia symptoms and that those with moderate or severe insomnia were more likely to have depressive symptoms compared with those without insomnia symptoms. This is consistent with the findings of previous studies performed during the COVID-19 pandemic that found that poor subjective sleep quality was associated with depressive symptoms in pregnant women (13). This could be because a tired brain can cause abnormal neuronal firing, leading to various neuropsychiatric symptoms (42). However, other studies have shown that the relationship between poor sleep quality and depressive symptoms is only evident in pregnant women aged above 30 years (43). This issue requires additional in-depth research in the future. In addition, insomnia is frequently associated with anxiety, adverse pregnancy outcomes, bed partner influence, and somatic discomfort (30), which in turn are frequently associated with the development of depression. However, sleep disturbances worsen as pregnancy progresses (44). Therefore, monitoring the sleep quality of pregnant women, especially during the third trimester, and formulating targeted interventions to improve insomnia symptoms are urgently needed. In addition, anxiety is prevalent among pregnant women, with the prevalence of prenatal anxiety ranging from 7.5 to 54.0% (39, 45). The proportion of women with anxiety during late pregnancy in our study was 20.2%. A previous study has reported that anxiety in pregnant women can directly and indirectly affect depression and that numerous factors influence depression through anxiety (1). The results of the current study are consistent with those of previous studies showing that maternal anxiety symptoms are associated with depressive symptoms during late pregnancy. Therefore, the prevention, early recognition, and interventions of anxiety in pregnant women are crucial. Altered environmental conditions, such as the COVID-19 pandemic, increase the risk of developing mental disorders (14). This study showed that pregnant women who experienced a high fear of COVID-19 exhibited higher rates of depressive symptoms, consistent with the results of a study performed in Italy (11). Pregnant women with a high fear of COVID-19 tend to spend considerable time paying excessive attention to news related to COVID-19 and its serious impact on the body, inevitably increasing the risk of depressive symptoms. This fear of pregnant women can be reduced by providing them with more knowledge about COVID-19 and pregnancy. Great attention should be paid to the problems related to pregnant women caused by such public emergencies.

### Effect of social factors on depression

It is worth noting that the increased pressure on the healthcare system caused by the COVID-19 pandemic may have resulted in limited access to healthcare services for pregnant women. A previous study showed that 82.4% of pregnant women believed that the pandemic had a negative impact on pregnancy monitoring (8). Our study found that 34.4% of pregnant women had delayed prenatal care during the pandemic, and that those who received delayed pregnancy care were more likely to have depressive symptoms, which is similar to previous studies. Previous studies have shown that concerns about poor care during pregnancy were significantly associated with elevated depressive symptoms during the pandemic (27). Moreover, it has been shown that changes in perinatal health practices, such as canceling appointments, may lead to increased levels of perinatal mental distress (46). Therefore, under public health emergencies, such as COVID-19, greater attention should be paid to the routine prenatal care of pregnant women, while the content related to emergency response should be increased. Noise is among the important environmental risk factors that have harmful effects on human health. Higher residential noise exposure is associated with increased odds of prenatal depression. High noise exposure increased the odds of prenatal depression by 71% compared with low levels of noise exposure (45). In the univariate analysis, we also found that pregnant women who reported the presence of noise in their place of residence had significantly higher rates of depressive symptoms vs. those who did not. A strong association between noise exposure and annoyance has been reported (47). In turn, noise annoyance has been linked to sleep disturbances, which can increase negative emotions and lead to depression (48). In addition, previous studies have shown that ambient noise and air pollution often co-occur (49). Because of the limitation of the conditions, data on environmental pollution could not be obtained in this study, and may be worth including in future studies. Food insecurity has been found to be associated with higher odds of prenatal depression (10). However, our study found that pregnant women who reported having a good drinking water quality at home were more likely to be protected from depression. This is similar to previous findings in other populations, in which household water insecurity was associated with postpartum depression (50). With the development of economies and the improvement of people's requirements for quality of life, the quality of the drinking water has become an increasingly issue of concern. Therefore, improving the quality of the drinking water for residents should be a priority, as it is not only beneficial for physical health, but also contributes to the improvement of mental health. Moreover, adequate family support during pregnancy is one of the important protective factors against depression (51). Talking to family members and family activities were associated with lower depressive symptoms in pregnant women (28). This study also found that family dysfunction was associated with depressive symptoms. Pregnant women with moderate or severe family dysfunction in late pregnancy were more likely to have depressive symptoms than were those with a high family function. In addition, pregnant women with good family functions receive more family support, which can help them deal with and endure various minor problems during pregnancy, thus minimizing the risk of depression. Family dysfunction can impart to pregnant women a feeling of a lack of care and support, that problems and troubles cannot be solved and eliminated in time, thus increasing the possibility of depression. Based on the effect of family dysfunction on depressive symptoms among pregnant women, a heightened communication between pregnant women and their families should be encouraged. Concomitantly, health-related education should also be provided to family members (39). Greater social support for pregnant women may be a viable way to reduce depressive symptoms in this population (18). This study found that poor social support was associated with maternal depressive symptoms in late pregnancy. This is similar to previous findings that poor social support is significantly associated with anxiety in pregnant women (52). Moreover, poor social support can lead to a lack of information, tools, and emotional sources, and can make pregnant women feel isolated and lonely, which can predispose them to depressive symptoms. Therefore, the strategies adopted in interventions for depressive symptoms in pregnant women should include the improvement of poor social support.

### Limitations

Although this study had important theoretical and practical implications, it had several limitations that warrant discussion. First, this study was a cross-sectional survey, and the causal relationship between the variables could not be obtained; therefore, longitudinal research is needed in the future. Second, this study was conducted in a city located in the northeast of Jiangsu Province, Eastern China, which may only represent areas with similar characteristics, thus limiting the generalizability of the findings. Third, this study focused on women in the late pregnancy stage, while pregnant women in other stages of pregnancy were not recruited; therefore, future studies should broaden the study population and increase the sample size. Finally, the variables assessed in this study were measured via self-reports, potentially leading to a bias. In addition, the PHQ-9 scale alone was used to measure depression in this study; thus, other measures of depression should be applied in the future for comparison. Despite these limitations, the current study contributes to the understanding of maternal depression during late pregnancy.

## Conclusion

The prevalence of maternal depressive symptoms during late pregnancy was high in Eastern China. Therefore, maternal healthcare providers should raise awareness regarding maternal depression symptoms during late pregnancy. Moreover, screening and effective interventions should be developed to alleviate these symptoms. Attempts should be made to alleviate symptoms of insomnia and anxiety, COVID-19-related fear, and delays in prenatal care. Moreover, efforts should be made to improve drinking water quality and address issues related to family dysfunction and poor social support. Concomitantly, greater attention should be paid to multiparous women to detect depressive symptoms in a timely manner and reduce the adverse consequences of maternal depression on the individual, child, family, and society.

## Data availability statement

The original contributions presented in the study are included in the article/supplementary material, further inquiries can be directed to the corresponding authors.

## Ethics statement

The studies involving human participants were reviewed and approved by the Ethics Committee of Lianyungang Maternal and Child Health Hospital. The patients/participants provided their written informed consent to participate in this study.

## Author contributions

XC, QM, and TZ conceived, designed, and advanced the entire study. XC analyzed the data and wrote the manuscript. All authors read, revised, and approved the final manuscript and agree to be accountable for all aspects of the work.
